# Plasma proteome changes associated with refractory cytopenia with multilineage dysplasia

**DOI:** 10.1186/1477-5956-9-64

**Published:** 2011-10-05

**Authors:** Pavel Májek, Zuzana Reicheltová, Jiří Suttnar, Jaroslav Čermák, Jan E Dyr

**Affiliations:** 1 Department of Biochemistry, Institute of Hematology and Blood Transfusion, Prague, Czech Republic

**Keywords:** myelodysplastic syndrome, refractory cytopenia, dysplasia, proteome

## Abstract

**Background:**

Refractory cytopenia with multilineage dysplasia (RCMD) is a subgroup of myelodysplastic syndrome (MDS), which belongs to oncohematological diseases, occurring particularly in elderly patients, and represents a heterogeneous group of bone marrow diseases. The goal of this study was to look for plasma proteins that changed quantitatively or qualitatively in RCMD patients.

**Results:**

A total of 46 plasma samples were depleted, proteins were separated by 2D SDS-PAGE (pI 4-7), and proteomes were compared using Progenesis SameSpots statistical software. Proteins were identified by nanoLC-MS/MS. Sixty-one unique, significantly (p < 0.05, ANOVA) different spots were found; proteins in 59 spots were successfully identified and corresponded to 57 different proteins. Protein fragmentation was observed in several proteins: complement C4-A, complement C4-B, inter-alpha-trypsin inhibitor heavy chain H4, and endorepellin.

**Conclusions:**

This study describes proteins, which change quantitatively or qualitatively in RCMD patients, and represents the first report on significant alterations in C4-A and C4-B complement proteins and ITIH4 fragments in patients with MDS-RCMD.

## Background

Refractory cytopenia with multilineage dysplasia (RCMD) is a subgroup of myelodysplastic syndrome (MDS). MDS itself belongs to the group of oncohematological diseases, occurring particularly in elderly patients. It represents a heterogeneous group of bone marrow diseases characterized by blood cytopenias, ineffective hematopoiesis, and dysplasia in one or more blood cell lines. According to the WHO (World Health Organization) classification of MDS, RCMD is defined by the presence of bicytopenia or pancytopenia in peripheral blood and dysplastic changes that are present in 10% or more of the cells in two or more myeloid lineages in the bone marrow (with less than 15% ringed sideroblasts) [1].

Despite the efforts and development in MDS research within the last several years, the pathogenesis of MDS remains still unclear. Several studies have shown an up- or down-regulation of different groups of genes in MDS patients [2-6]; however, the results are in some cases controversial and difficult to interpret. Proteomic techniques might provide a new and more detailed set of information clarifying the molecular mechanisms involved in the development of MDS [7]. Complex protein-protein networks reflect the changes at the transcription level, as well as changes induced by protein modifications depending on (patho) physiological conditions in organisms. Posttranslational modifications of proteins including fragmentation or cross-linking are examples of changes detected exclusively by proteomic techniques [8], which may play a crucial role in the development and progression of the disease [9,10].

Two-dimensional gel electrophoresis (especially 2D SDS-PAGE) is one of the most widespread proteomic techniques. Despite some disadvantages like the co-identification of proteins within a protein feature, a limited range of detection of low-abundant proteins, or problems with the analysis of basic and low molecular weight proteins, 2D electrophoresis offers both the possibilities to search for protein level changes and for protein posttranslational modifications. Differential proteome pattern analysis, based on either quantitative or qualitative changes, combined with the identification of proteins by mass spectrometry, enables a deeper insight into the changing proteomes.

The aim of this study was to identify both quantitative and qualitative changes in plasma proteomes of MDS patients with RCMD using 2D electrophoresis.

## Methods

A total of 22 RCMD patient (11 males and 11 females) plasma samples and 24 healthy controls (10 males and 14 females) have been investigated. The diagnosis of RCMD was established according to the WHO classification criteria [1]. The age of the patients ranged from 21 to 72 years. Healthy control ages ranged from 20 to 36 years. All individuals tested agreed to participate in the study on the basis of an informed consent. All samples were obtained and analyzed in accordance with the Ethical Committee regulations of the Institute of Hematology and Blood Transfusion.

Blood samples were collected by venipuncture into tubes coated with EDTA. Plasma was obtained by the centrifugation (5 min, 4000 × g) of blood samples; and plasma aliquots were then transferred to polypropylene Eppendorf tubes and stored at -70°C until used. Thawed plasma samples were centrifuged (5 min, 12000 × g), diluted 1:3 by depletion buffer (Agilent, Santa Clara, CA, USA) and filtrated using 0.22 μm Spin filters (Agilent,) for 1 minute by centrifugation at 12000 × g. A MARS Hu-14 4,6 × 100 mm column (Agilent) was used to deplete fourteen high-abundant proteins (albumin, IgG, antitrypsin, IgA, transferrin, haptoglobin, fibrinogen, alpha-2-macroglobulin, alpha-1-acid glycoprotein, IgM, apolipoprotein AI, apolipoprotein AII, complement C3 and transthyretin). 5K MWCO Spin Concentrators (Agilent) were used to desalinate and concentrate the samples (3000 × g, 20°C). MilliQ water (4 mL) was added to concentrated samples and the desalinating-concentrating step was repeated three times. Finally, desalinated and concentrated samples were vacuum dried, frozen rapidly, and stored at -70°C.

1D and 2D SDS-PAGE, image analysis, protein digestion, and mass spectrometry analysis were performed as described previously [8,11,12]. Briefly, isoelectric focusing (IPG strips pI 4-7, 7.7 cm) and SDS-PAGE (8 × 10 cm, 10% resolving gel, 3.75% stacking gel, 5°C, 30 mA/gel) were used in the first and the second dimensions, respectively. Gels were stained with colloidal Coomassie blue, scanned, and processed with Progenesis SameSpots software (Nonlinear Dynamics, Newcastle upon Tyne, UK), which computed multiplication (fold) and p-values of all spots using one-way ANOVA analysis. PCA (Principal Component Analysis) was performed to assess whether grouping of patients and healthy controls based on proteomic methods reflects their stratification using classical clinical diagnosis. PCA was performed making use of the same software focusing only on the spots of statistical significance employed for protein identification.

Selected spots were excised from the gel, and proteins were in-gel digested by trypsin. An HCT ultra ion-trap mass spectrometer (Bruker Daltonics, Bremen, Germany) with nanoelectrospray ionization, coupled to a UltiMate 3000 nanoLC system (Dionex, Sunnyvale, CA, USA) was used for mass spectrometry analysis. Proteins were identified using tandem mass spectrometry. Mascot (Matrix Science, London, UK) was used for database searching (SWISS-PROT release 2010_12). Two unique peptides (fulfilling a minimal Mascot score) were necessary to successfully identify a protein. Two cleaning runs were performed before and after each sample run to eliminate peptide carry-over between nanoLC separations. The N-terminal tryptic peptide of the endorepellin LG3 fragment corresponding to ^4197^DAPGQYGAYFHDDGFLAFPGHVFSR^4221 ^was monitored (z = +2, precursor ion 1387.0 m/z, selection window width = 2) as described in previous literature [13].

Western blotting was performed as described previously [8]. Proteins were transferred from gel to a PVDF membrane (10 V constant voltage for 1 hr); the membrane was then incubated with a blocking buffer (3% BSA in PBS) at 4°C overnight, rinsed; and incubated with primary antibodies: anti-ITIH4 (Abnova, Taipei, Taiwan), 1:2000 dilution, at 30°C for 45 min, or anti-HSPG2 (LifeSpan BioScience, Seattle, WA, USA), 1:1000 dilution, at 30°C for 45 min. Then the membrane was incubated with the secondary antibody, rabbit anti-mouse IgG antibody conjugated with peroxidase (Sigma-Aldrich, Prague, Czech Republic), 1:60000 dilution, at 30°C for 45 min. After rinsing, a chemiluminescent substrate (SuperSignal West Pico; Thermo Scientific, Waltham, MA, USA) was added to the membrane for 5 min; and an appropriate film exposition (Amersham Hyperfilm ECL; GE Life Sciences, Piscataway, NJ, US) was performed.

Complement C4a des Arg plasma level was measured using a commercial EIA kit (Enzo Life Sciences, Farmingdale, NY, USA). Samples were measured in duplicate according to manufacturer instructions. Results were expressed as means ± standard deviations. An unpaired t-test was used for comparison of complement C4a des Arg plasma levels when the RCMD group was compared with the control group.

## Results

**Figure 1:**
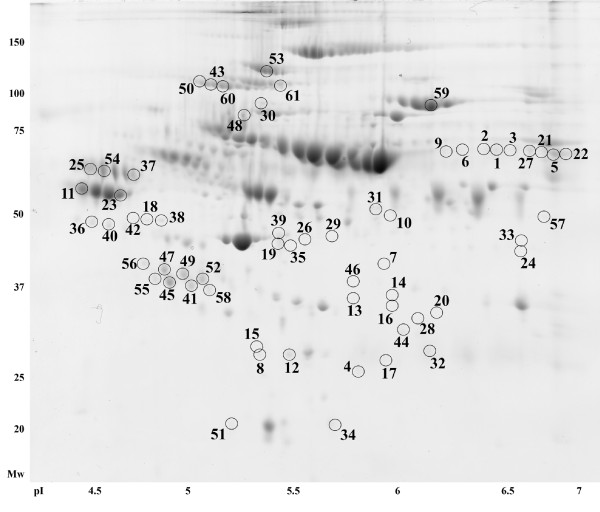
**Positions of significantly differed spots on a 2D gel**. Positions of all spots that were found to significantly differ in 2D gels of RCMD patients and healthy controls when mutually compared. The 2D gel of a patient sample was used as an illustrative gel to display spot positions.

2D gels were prepared for the experiment, and scanned 2D gel images were divided into RCMD (n = 22) and the control group (n = 24). Comparing these two groups we found 61 unique spots that differed significantly (p < 0.05) in normalized volumes (Figure 1). Proteins in 59 spots were successfully identified, which correspond to 57 different proteins. The list of all spots including ANOVA p-values, the multiplication (fold value), protein identification with the number of identified peptides (unique peptides above the threshold score), protein accession number (Swiss-Prot), the sequence coverage, both calculated (theoretical) and experimental values of pI and molecular weight, is summarized in Table S1 (See additional file 1: Table S1).

**Figure 2:**
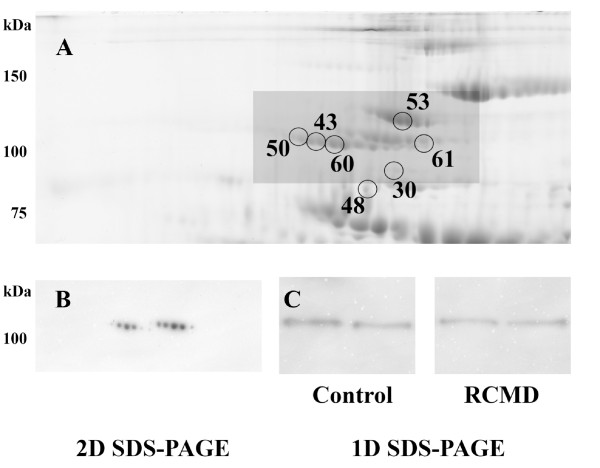
**ITIH4 western blot analysis**. (A) A segment of 2D SDS-PAGE gel containing spots with uncleaved ITIH4 (spots 43, 50, 53, 60, and 61) and (B) 2D western blot analysis (ITIH4) - a segment corresponding to the highlighted area on the 2D gel; (C) illustrations of 1D western blot analysis of ITIH4 and its bands in both the control and RCMD groups corresponding to the molecular weight of 2D western blot (and 2D SDS-PAGE) ITIH4 spots are shown.

Protein fragmentation was observed in several proteins, when comparing protein molecular weights estimated on 2D maps with known or predicted values (complement C4-A, complement C4-B, inter-alpha-trypsin inhibitor heavy chain H4, endorepellin). Combining the 2D electrophoresis data with mass spectrometry results, several fragments were identified. Both complement C4-A and C4-B were identified in 17 spots with molecular weights ranging from around 20 to 100 kDa. Sequences of the identified peptides corresponded to the complement C4-A(B) fragments, as well as their molecular weights: complement C4 beta chain, complement C4-A alpha chain, complement C4-B alpha chain, complement C4 gamma chain, C4b-A, C4b-B, C4d-B, and C4c. Most of the spots with identified complement C4-A(B) had their normalized volumes increased in RCMD, when compared to the control group. Although co-identification of other proteins within a spot confused interpretation of the results, there were several spots (12, 15, 24, and 33) with unique identification of fragments; these spots corresponded to the C4c fragment (spot 12 and 15) and C4 gamma chain (spot 24 and 33). Normalized volumes of all of these spots were increased in RCMD (1.9 and 1.8 fold for C4c, 1.7 and 1.5 fold for C4 gamma); and thus showed that complement C4-A(B) fragmentation or fragment modifications were present in RCMD patients at a higher rate. An inter-alpha-trypsin inhibitor heavy chain H4 (ITIH4) was identified in 13 spots, with estimated molecular weights of about 35 and 100 kDa (except spot 48). These molecular weights corresponded to 35 kDa ITIH4 fragments, and to an uncleaved ITIH4 (120 kDa). The estimated molecular weight of spot 48 was about 80 kDa and probably corresponded to 70 kDa ITIH4 fragment, however, this band was not observed when western blot analysis was performed. Sequences of peptides identified by MS/MS corresponded to these polypeptides, in agreement with the electrophoresis data. In spite of the co-identification of other proteins within the spots with an identified ITIH4, there was an obvious trend in the changes between RCMD and the control group. All spots with ITIH4 fragments (spots 41, 45, 46, 48, 49, 52, 55, and 58) had the normalized volumes decreased in RCMD, while normalized volumes of spots with unprocessed (uncleaved) ITIH4 were increased in RCMD, when compared with the control group. Identification of ITIH4 (ucleaved protein containing spots 43, 50, 53, 60, and 61) was validated by 2D SDS-PAGE western blotting (Figure 2). ITIH4 expression was assessed by western blot analysis: a single band of more than 100 kDa molecular weight was observed and corresponded to the uncleaved ITIH4. No difference in ITIH4 expression between the patient group (n = 8) and the control group (n = 8) was observed (Figure 2). Using monoclonal anti-ITIH4 antibody capable of detecting uncleaved ITIH4 and 70 kDa ITIH4 fragment we did not observe the 70 kDa ITIH4 fragment (the antibody was not against the 35 kDa ITIH4 fragments). Basement membrane-specific heparan sulfate proteoglycan core protein (spot 34), known as perlecan, fragmentation was observed in RCMD with a difference of 1.5 fold, when compared with the control group. All identified peptide sequences corresponded to the perlecan fragment endorepellin (3687-4391 amino acid sequence of perlecan) or more precisely to the perlecan/endorepellin fragment LG3 (4197-4391 amino acid sequence of perlecan). The sequences of three peptides fulfilling a minimal Mascot score for identity were as follows: ^4236^TSTASGLLLWQGVEVGEAGQGK^4257^, ^4258^DFISLGLQDGHLVFR^4272^, and ^4330^GSVYIGGAPDVATLTGGR^4347 ^(See additional file 2: Figure S1). One additional peptide fulfilled a minimal Mascot score for homology: ^4222^SLPEVPETIELEVR^4235^. The fragmentation of perlecan was further monitored by western blotting (n = 8, each group) but no bands or spots (using 1D or 2D western blot) corresponding to the position (or Mw) of the spot 34 were observed.

**Figure 3:**
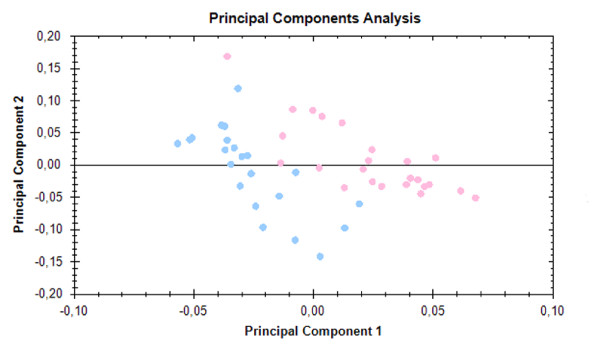
**Principal Component Analysis**. PCA was performed to assess whether grouping of patients and healthy controls based on proteomic methods reflects their stratification using classical clinical diagnosis. Analysis was based on spots that significantly differ according the mentioned statistical criteria (p < 0.05, ANOVA). Principal Component Analysis (PCA) showed the separation of all samples into two aggregates that corresponded to the RCMD (blue dots) and the control group (pink dots).

PCA was performed to assess whether grouping of patients and healthy controls based on proteomic methods reflects their stratification using classical clinical diagnosis. Analysis was based on spots that significantly differ according the mentioned statistical criteria (p < 0.05, ANOVA). PCA showed an obvious separation of all samples (aligned gel images) into two aggregates that corresponded to the RCMD and the control group, respectively (Figure 3).

The plasma level of complement C4a des Arg was measured in both control and patient groups: 5.8 ± 3.1 μg/mL (the control group, n = 19) and 6.4 ± 9.6 μg/mL (the RCMD group, n = 19). No significant difference was observed between these two groups (t-test, p = 0.83).

## Discussion

In this study we present data characterizing changes in the plasma proteome of patients with RCMD. Most of the identified changes are related to the complement C4-A and C4-B proteins and to the fragmentation of several plasma proteins. Among all the identified proteins, albumin was observed in the largest number of spots (26 spots in total). Although the immunodepletion column was used to deplete high-abundant plasma proteins, several of them were identified or co-identified within many spots (albumin, haptoglobin, fibrinogen, apolipoprotein AI and complement C3). This is probably a result of posttranslational modifications or fragmentation of these proteins, which might have affected the process of immunodepletion by influencing the binding of the proteins to stationary phase of the depletion column. In the case of albumin, there was always a decrease in the spot normalized volume in RCMD when this protein was identified uniquely within a spot (2, 7, 17, 20 etc.). This suggests that the albumin modification was not caused by RCMD (it was present in healthy control plasma samples), the decrease in the spot volume might be caused by acute phase reaction. The other four high-abundant plasma proteins were not uniquely identified within their spots. At present it is hard to speculate about the possible impact of this finding.

Our study demonstrates alterations in C4-A and C4-B complement proteins. The fragmentation, or modification of C4 fragments of both proteins was observed with an approximately 2 fold (C4c fragment) and 1.6 fold (C4 gamma) increase in the RCMD group, when compared to the healthy controls. C4c and C4 gamma were the only C4 (or any other complement) protein fragments uniquely identified within a spot. Moreover, we detected all possible fragments of C4-A(B) produced by processing of their precursors (described in *Results*) as non-unique identifications. The plasma level of complement C4a des Arg was measured in both control and patient groups. There was higher variability of concentration values in RCMD patients when compared to the control group but no statistically significant difference in the plasma level between the two groups was observed. In view of the fact that the results obtained by electrophoresis showed two-fold increase of normalized spot volumes in the RCMD group with low variability (p-values of spots 8, 12 or 15 less than 0.001) we suppose that the observed changes were not associated with complement activation. This assumption is also supported by the fact that no changes in other complement proteins were observed. Our result may be explained by a different role of C4-A(B) fragments or by a different cause of fragmentation in RCMD patients [10]. The immune system plays substantial and diverse roles in the development of cancer; it may either eliminate the initiation of cancer development or facilitate cancer progression [14,15]. The complement system, as a part of the immune system, participates in immune response; with it having been shown recently that the C4d fragment may be of clinical interest in the case of transplantations and consequential rejections [16-18]. Although the most expected cause of complement activation would be infection, it has been recently shown that complement activation products are also involved in promoting tumor growth [14,19]. Experimental studies have shown that tumor growth was greatly reduced in mice deficient in the complement protein C4 [14]. It is also known that MDS patients frequently exhibit multiple abnormalities in the expression of neutrophil complement receptors and granular components (>3), as well as in other cell functions, suggesting the possibility of using these phenotypic abnormalities in the monitoring of disease progression [20].

Inter-alpha-trypsin inhibitor heavy chain H4 (ITIH4) is involved in acute phase reaction and has been found to be a possible cancer marker [21]. ITIH4 fragments have been found to be potentially associated with MDS [9]; and several authors have shown differences in the fragmentation of ITIH4 [21,22]. Furthermore, an alternation in ITIH4 fragments has been described in several malignant diseases [22-25]. In our experiments, we were able to divide all spots containing ITIH4 into two groups according to their molecular weight (described in *Results*), which corresponded to ITIH4 fragments (probably produced by kallikrein) and to the uncleaved protein. In spite of co-identification of other proteins in spots containing ITIH4, a trend has been observed in our MDS patients: normalized volumes of all spots that contained uncleaved ITIH4 were increased in the RCMD group; while normalized volumes of all spots with fragmented ITIH4 were decreased in RCMD patients. Such a difference could be explained by further ITIH4 fragmentation producing smaller fragments and peptides, which could not be detected by 2D SDS-PAGE as designed in our study, or a reduced fragmentation rate of uncleaved ITIH4 in MDS patients. To assess whether the observed changes could be caused by changed ITIH4 expression (possible influence of the acute phase reaction), western blot analysis of ITIH4 was performed. We observed no differences between the RCMD and control groups, therefore the increase in spot normalized volumes of uncleaved ITIH4 (as mentioned above) was evidently caused by proteins co-identified within the same spot(s). As the ITIH4 expression was not changed, the alteration of its fragments could be caused by a different fragmentation (further processing of the fragments) as it was suggested in the literature [23,24]. It is also possible that the fragmentation level was not changed and the differences observed in the ITIH4 fragment spots were caused by posttranslational modification(s) or by combination of both the factors.

Basement membrane-specific heparan sulfate proteoglycan core protein, also known as perlecan, is an integral component of basement membranes. This protein, identified in spot 34, is cleaved and produces endorepellin, which has anti-tumor and anti-angiogenic properties [26]. Perlecan (or endorepellin) can be further processed by bone morphogenic protein-1 (BMP-1) to produce LG3 peptide, which also has anti-angiogenic properties [13,27,28]. All peptides identified in spot 34 belonged not only to perlecan or to its fragment endorepellin, but all peptide sequences corresponded to LG3 peptide. As BMP-1 cleaves endorepellin specifically between 4196Asn and 4197Asp [28], trypsin digest of LG3 produces the N-terminal tryptic peptide (^4197^DAPGQYGAYFHDDGFLAFPGHVFSR^4221^) containing the N-terminal non-tryptic site. Identification of this peptide in the sample could prove whether it is LG3 peptide produced by the specific proteolytic cleavage of endorepellin by BMP-1, or just an endorepellin fragment corresponding to the LG3 sequence. As this N-terminal LG3 peptide was not identified we cannot exclude any possibility. The normalized volume of spot 34 containing the LG3 peptide or its fragment was increased in RCMD by 50%. Alterations of the LG3 peptide were observed in the plasma of breast cancer patients (decreased level) [13], in amniotic fluid of pregnant women with premature rupture of the fetal membrane [29], or in the urine of end-stage renal failure patients [30]. The alteration of an endorepellin fragment in RCMD, whether it is due to a plasma level increase or posttranslational modification, may be connected to its anti-tumor and anti-angiogenic properties. This may be supported by the fact that endorepellin has been shown to inhibit tumor growth and cancer cell metabolism *in vivo *and has been proposed to be a possible anti-tumor agent [26]. To assess the fragmentation of endorepellin or the influence of the protein (or further protein fragments) plasma level changes to 2D SDS-PAGE based results western blot analysis was performed. However, we did not observe bands or spots (using 1D or 2D western blot) corresponding to the endorepellin fragment (spot 34). The change of spot 34 normalized volume could be also caused by the presence of other protein(s) but there was no other co-identified protein except perlecan in spite of repeated MS/MS analysis. Nevertheless, especially proteins of lower Mw (or some glycoproteins etc.) might cause difficulty in identification so we cannot exclude the possibility of co-localized protein(s) within the spot 34.

An important issue that should be discussed is the choice of the control group used. As MDS usually occurs in elderly patients the optimal control group should match for age as well as for sex. However, matching the control group for age may be connected with some difficulties. It is a good assumption that there may occur some proteome changes related to ageing. A paper by Ignjatovic et al. [31] points at plasma proteome differences when compared neonates, children and adults. Consequently, we suppose that there are some differences between our RCMD patient group and the control group because of different age ranges. However, collecting a control group of healthy individuals of older age is a problem as there are some conditions that have to be fulfilled. The main issue is to find healthy individuals of older age, who do not suffer from other diseases (blood pressure, diabetes etc.). In spite of collecting such samples, we suppose it will be necessary to use more control samples of such group to suppress possible biological variations. Considering that, we decided to compare our patient group with a "really" healthy control group as used in the manuscript. As it is the first proteomic study of RCMD patients we wanted to see all the changes that would occur. Moreover, there is a couple of MDS patients of young age (in case of RCMD from 21 years) that should be compared too. Nevertheless, it is the future task to compare other control groups with MDS. Much more difficult task will be to assess the influence of ageing in "healthy" population on the plasma proteome.

## Conclusions

In conclusion, this study represents the first report on the significant alterations in C4-A and C4-B complement proteins and in ITIH4 fragments in patients with MDS-RCMD. Our results show the involvement of complement system proteins and protein fragmentation in most of the proteome changes identified in this study. However, a more extensive study will be needed to compare the results obtained from RCMD patients with patients from other MDS subgroups. These studies hopefully should help us to establish a possible role of the altered activation (modification) of several parts of complement, the different fragmentation of ITIH4 and the endorepellin fragment in the promotion of the pathological clone in MDS.

## Competing interests

The authors declare that they have no competing interests.

## Authors' contributions

PM and ZR designed and performed research, analyzed data and wrote the manuscript. JC diagnosed the patients and collected the samples. JS, JC and JED designed research and wrote the manuscript. All authors read and approved the final manuscript.

## Supplementary Material

Additional file 1 **Table S1**. List of spots that significantly differ when RCMD patients and healthy controls compared. Click here for file

Additional file 2 **Figure S1 - The peptide sequences corresponding to the perlecan fragment endorepellin**. An illustration of the coverage of perlecan sequence (shown in bold red) that corresponds to the perlecan fragment endorepellin (3687-4391 amino acid sequence of perlecan) or more precisely to the perlecan/endorepellin fragment LG3 (4197-4391 amino acid sequence of perlecan). The MS/MS spectra of three peptides fulfilling a minimal Mascot score for identity ^4236^TSTASGLLLWQGVEVGEAGQGK^4257^, ^4258^DFISLGLQDGHLVFR^4272^, and ^4330^GSVYIGGAPDVATLTGGR^4347 ^are shown. Click here for file
