# Supplementary material for: Plasma proteome changes associated with refractory cytopenia with multilineage dysplasia
Source: Proteome Sci. 2011 Oct 5;9:64. doi: 10.1186/1477-5956-9-64 (PMC3192726; doi:10.1186/1477-5956-9-64)
Supplement: Additional file 1 — Table S1. List of spots that significantly differ when RCMD patients and healthy controls compared. [file 1477-5956-9-64-S1.PDF]

Table 1. List of spots that significantly differ when RCMD patients and healthy controls compared.

							Calculated		Experimental	
spot	p	fold	protein	peptides	AC	SC (%)	pI	Mw (kDa)	pI	Mw (kDa)
1	0.000015	-2.8	Serum albumin	19	P02768	53	5.92	69	6.5	70
			Hemopexin	3	P02790	23	6.55	52		
2	0.000014	-2.7	Serum albumin	15	P02768	45	5.92	69	6.5	70
3	0.000024	-2.6	Serum albumin	19	P02768	46	5.92	69	6.6	70
			Hemopexin	2	P02790	12	6.55	52		
			Complement C4-A; (B)	4	P0C0L4;P0C0L5	9	6.66;6.73	193;193		
			Fibrinogen alpha chain	2	P02671	7	5.70	95		
4	0.000308	-2.4	Serum albumin	6	P02768	20	5.92	69	5.8	27
			Apolipoprotein A-I	4	P02647	28	5.56	31		
5	0.000071	-2.4	Serum albumin	20	P02768	68	5.92	69	6.8	70
			Fibrinogen alpha chain	5	P02671	12	5.70	95		
			Alpha-1-antichymotrypsin	2	P01011	11	5.33	48		
			Hemopexin	2	P02790	12	6.55	52		
			Complement component C8 beta chain	2	P07358	14	8.50	67		
6	0.000184	-2.4	Serum albumin	17	P02768	53	5.92	69	6.4	70
			Protein Z-dependent protease inhibitor	2	Q9UK55	9	8.28	51		
7	0.000011	-2.2	Serum albumin	11	P02768	25	5.92	69	6.0	38
8	0.000006	2.1	Complement C4-A; (B)	4	P0C0L4;P0C0L5	5	6.66;6.73	193;193	5.3	28
			Apolipoprotein A-I	3	P02647	26	5.56	31		
9	0.000516	-2.1	Serum albumin	20	P02768	61	5.92	69	6.3	70
			Protein Z-dependent protease inhibitor	2	Q9UK55	5	8.28	51		
			Hemopexin	2	P02790	12	6.55	52		
10	0.000127	-2.1	Serum albumin	10	P02768	31	5.92	69	6.0	50
			Complement factor I	2	P05156	11	7.72	66		
			Pigment epithelium-derived factor	2	P36955	6	5.97	46		
11	0.002	2.0	Alpha-1-antichymotrypsin	3	P01011	15	5.33	48	4.5	53
12	0.00054	1.9	Complement C4-A; (B)	4	P0C0L4;P0C0L5	6	6.66;6.73	193;193	5.5	28
13	0.048	1.9	Apolipoprotein E	4	P02649	36	5.65	36	5.8	33
			Serum albumin	2	P02768	17	5.92	69		
14	0.001	-1.9	unidentified						6.0	34
15	0.000023	1.8	Complement C4-A; (B)	2	P0C0L4;P0C0L5	3	6.66;6.73	193;193	5.3	29
16	0.000216	-1.8	unidentified						6.0	32
17	0.00059	-1.8	Serum albumin	3	P02768	11	5.92	69	6.0	28
18	0.000078	1.8	Complement C4-B	3	P0C0L5	5	6.73	193	4.8	48
			Serum paraoxonase/arylesterase 1	4	P27169	20	5.08	40		
19	0.018	1.7	Haptoglobin	4	P00738	16	6.13	45	5.4	40
			Apolipoprotein A-IV	3	P06727	12	5.28	45		
			Serum albumin	2	P02768	11	5.92	69		
20	0.000112	-1.7	Serum albumin	3	P02768	8	5.92	69	6.3	31
21	0.002	-1.7	Serum albumin	19	P02768	51	5.92	69	6.8	70
			Complement component C8 beta chain	2	P07358	14	8.50	67		
			Alpha-1-antichymotrypsin	2	P01011	7	5.33	48		
			Complement C4-A; (B)	4	P0C0L4;P0C0L5	5	6.66;6.73	193;193		
			Fibrinogen alpha chain	3	P02671	7	5.70	95		
22	0.009	-1.7	Serum albumin	15	P02768	45	5.92	69	6.9	70
			Alpha-1-antichymotrypsin	4	P01011	14	5.33	48		
			Fibrinogen alpha chain	4	P02671	7	5.70	95		
			Complement component C9	2	P02748	4	5.43	63		
			Hemopexin	2	P02790	10	6.55	52		
23	0.000008	-1.7	Alpha-2-HS-glycoprotein	6	P02765	38	5.43	39	4.6	53
			Corticosteroid-binding globulin	4	P08185	37	5.64	45		
			Alpha-1-antichymotrypsin	2	P01011	8	5.33	48		
24	0.002	1.7	Complement C4-A; (B)	2	P0C0L4;P0C0L5	3	6.66;6.73	193;193	6.6	40
25	0.00001	1.6	Alpha-1-antichymotrypsin	10	P01011	57	5.33	48	4.5	65
			Corticosteroid-binding globulin	2	P08185	11	5.64	45		
26	0.001	-1.6	Serum albumin	14	P02768	34	5.92	69	5.6	42
			Apolipoprotein A-IV	2	P06727	7	5.28	45		
27	0.008	-1.6	Serum albumin	16	P02768	47	5.92	69	6.7	70
			Complement C4-A; (B)	6	P0C0L4;P0C0L5	8	6.66;6.73	193;193		
			Fibrinogen alpha chain	3	P02671	9	5.70	95		
			Hemopexin	3	P02790	15	6.55	52		
			Complement component C8 beta chain	2	P07358	11	8.50	67		
28	0.000996	-1.6	Serum albumin	2	P02768	12	5.92	69	6.2	31
29	0.000193	-1.6	Serum albumin	8	P02768	32	5.92	69	5.7	42
30	0.029	-1.6	Antithrombin-III	5	P01008	21	6.32	53	5.3	85
			Complement C4-A; (B)	8	P0C0L4;P0C0L5	10	6.66;6.73	193;193		
			Vitamin D-binding protein	4	P02774	29	5.40	53		
			Plasma protease C1 inhibitor	5	P05155	15	6.09	55		
			Insulin-like growth factor-binding protein complex acid labile subunit	6	P35858	18	6.33	66		
			Ceruloplasmin	5	P00450	12	5.44	122		
			Alpha-1B-glycoprotein	3	P04217	12	5.56	54		
			Histidine-rich glycoprotein	3	P04196	7	7.09	60		
31	0.007	-1.5	Serum albumin	10	P02768	30	5.92	69	5.9	50
			Pigment epithelium-derived factor	8	P36955	22	5.97	46		
			Complement factor I	3	P05156	10	7.72	66		
			Histidine-rich glycoprotein	3	P04196	7	7.09	60		
32	0.019	-1.5	Serum albumin	2	P02768	12	5.92	69	6.3	28
33	0.013	1.5	Complement C4-A; (B)	2	P0C0L4;P0C0L5	4	6.66;6.73	193;193	6.6	42
34	0.045	1.5	Basement membrane-specific heparan sulfate proteoglycan core protein	3	P98160	1	6.06	469	5.7	20
35	0.023	-1.5	Serum albumin	9	P02768	33	5.92	69	5.5	40
			Apolipoprotein A-I	2	P02647	14	5.56	31		
			Apolipoprotein A-IV	2	P06727	16	5.28	45		
36	0.017	1.4	Leucine-rich alpha-2-glycoprotein	5	P02750	38	6.45	38	4.5	48
			Alpha-1-antichymotrypsin	2	P01011	8	5.33	48		
37	0.002	-1.4	Alpha-1-antichymotrypsin	14	P01011	41	5.33	48	4.7	65
			Kininogen-1	10	P01042	21	6.34	72		
			Alpha-2-HS-glycoprotein	3	P02765	28	5.43	39		
			Corticosteroid-binding globulin	4	P08185	19	5.64	45		
			Serum albumin	2	P02768	5	5.92	69		
38	0.008	1.4	Complement C4-B	3	P0C0L5	8	6.73	193	4.8	48
			Serum paraoxonase/arylesterase 1	3	P27169	23	5.08	40		
			Coagulation factor X	2	P00742	6	5.68	55		
			Zinc-alpha-2-glycoprotein	2	P25311	16	5.71	34		
39	0.009	1.3	Apolipoprotein A-IV	3	P06727	12	5.28	45	5.4	43
			Sex hormone-binding globulin	5	P04278	28	6.22	44		
			Actin, cytoplasmic 1	2	P60709	19	5.29	42		
			Haptoglobin	2	P00738	11	6.13	45		
			Serum albumin	2	P02768	6	5.92	69		
			Gamma-glutamyl hydrolase	2	Q92820	9	6.66	36		
			CD5 antigen-like	2	O43866	10	5.28	38		
40	0.043	1.3	Leucine-rich alpha-2-glycoprotein	4	P02750	23	6.45	38	4.6	48
41	0.000119	-1.3	Clusterin	3	P10909	15	5.88	52	5.0	35
			Inter-alpha-trypsin inhibitor heavy chain H4	2	Q14624	5	6.51	103		
42	0.039	1.3	Complement C4-B	3	P0C0L5	6	6.73	193	4.7	48
			Serum paraoxonase/arylesterase 1	3	P27169	19	5.08	40		
43	0.005	1.3	Inter-alpha-trypsin inhibitor heavy chain H4	17	Q14624	34	6.51	103	5.0	110
			Inter-alpha-trypsin inhibitor heavy chain H1	4	P19827	13	6.31	101		
			Phosphatidylinositol-glycan-specific phospholipase D	3	P80108	5	5.91	92		
44	0.006	1.3	Complement factor H-related protein 2	2	P36980	12	6.00	31	6.1	30
			Prostaglandin-H2 D-isomerase	2	P41222	17	7.66	21		
45	0.000346	-1.3	Clusterin	4	P10909	14	5.88	52	4.8	36
			Inter-alpha-trypsin inhibitor heavy chain H4	4	Q14624	9	6.51	103		
			Histidine-rich glycoprotein	2	P04196	4	7.09	60		
46	0.029	-1.3	Serum albumin	11	P02768	23	5.92	69	5.8	36
			Inter-alpha-trypsin inhibitor heavy chain H4	2	Q14624	4	6.51	103		
47	0.000364	-1.3	Clusterin	7	P10909	31	5.88	52	4.8	37
48	0.044	-1.3	Complement C4-A	14	P0C0L4	16	6.66	193	5.2	80
			Complement C4-B	14	P0C0L5	16	6.73	193		
			Angiotensinogen	6	P01019	26	5.87	53		
			Vitamin D-binding protein	7	P02774	32	5.40	53		
			Antithrombin-III	5	P01008	16	6.32	53		
			Insulin-like growth factor-binding protein complex acid labile subunit	5	P35858	19	6.33	66		
			Afamin	6	P43652	15	5.64	69		
			Alpha-1B-glycoprotein	5	P04217	19	5.56	54		
			Plasma protease C1 inhibitor	4	P05155	13	6.09	55		
			Ceruloplasmin	4	P00450	11	5.44	122		
			Inter-alpha-trypsin inhibitor heavy chain H4	3	Q14624	6	6.51	103		
			Complement component C9	2	P02748	8	5.43	63		
49	0.000018	-1.3	Clusterin	6	P10909	21	5.88	52	4.9	37
			Inter-alpha-trypsin inhibitor heavy chain H4	5	Q14624	13	6.51	103		
			Complement C3	4	P01024	4	6.02	187		
50	0.018	1.2	Inter-alpha-trypsin inhibitor heavy chain H4	16	Q14624	33	6.51	103	5.0	115
			Inter-alpha-trypsin inhibitor heavy chain H1	11	P19827	23	6.31	101		
			Inter-alpha-trypsin inhibitor heavy chain H2	7	P19823	14	6.40	106		
			Phosphatidylinositol-glycan-specific phospholipase D	9	P80108	20	5.91	92		
			Complement C4-A; (B)	4	P0C0L4;P0C0L5	6	6.66;6.73	193;193		
			Angiotensinogen	2	P01019	12	5.87	53		
			Alpha-1-antichymotrypsin	2	P01011	7	5.33	48		
			Complement C5	3	P01031	4	6.11	188		
			Kininogen-1	3	P01042	13	6.34	72		
			Plasma protease C1 inhibitor	2	P05155	7	6.09	55		
			Antithrombin-III	2	P01008	10	6.32	53		
			Inter-alpha-trypsin inhibitor heavy chain H3	2	Q06033	2	5.49	100		
51	0.011	1.2	Retinol-binding protein 4	2	P02753	26	5.76	23	5.2	20
			Ferritin light chain	2	P02792	25	5.50	20		
52	0.001	-1.2	Clusterin	5	P10909	17	5.88	52	5.0	36
			Inter-alpha-trypsin inhibitor heavy chain H4	5	Q14624	7	6.51	103		
			Complement C3	4	P01024	6	6.02	187		
			Antithrombin-III	3	P01008	11	6.32	53		
53	0.008	1.2	Ceruloplasmin	25	P00450	49	5.44	122	5.3	125
			Antithrombin-III	5	P01008	23	6.32	53		
			Inter-alpha-trypsin inhibitor heavy chain H4	7	Q14624	16	6.51	103		
			Vitamin D-binding protein	3	P02774	18	5.40	53		
			Inter-alpha-trypsin inhibitor heavy chain H3	2	Q06033	10	5.49	100		
			Alpha-1B-glycoprotein	2	P04217	16	5.56	54		
			Angiotensinogen	2	P01019	8	5.87	53		
54	0.009	1.2	Alpha-1-antichymotrypsin	12	P01011	58	5.33	48	4.6	65
			Kininogen-1	4	P01042	13	6.34	72		
			Corticosteroid-binding globulin	2	P08185	11	5.64	45		
55	0.037	-1.2	Clusterin	5	P10909	15	5.88	52	4.8	36
			Inter-alpha-trypsin inhibitor heavy chain H4	4	Q14624	5	6.51	103		
			Histidine-rich glycoprotein	2	P04196	4	7.09	60		
56	0.042	-1.2	Clusterin	4	P10909	18	5.88	52	4.7	38
			Histidine-rich glycoprotein	3	P04196	7	7.09	60		
57	0.039	1.2	Carboxypeptidase N catalytic chain	4	P15169	19	6.86	52	6.8	50
			Plasma serine protease inhibitor	6	P05154	24	9.30	46		
			Plasma kallikrein	2	P03952	11	8.60	71		
			Complement C4-A; (B)	4	P0C0L4;P0C0L5	5	6.66;6.73	193;193		
			Pigment epithelium-derived factor	2	P36955	13	5.97	46		
			Beta-2-glycoprotein 1	3	P02749	30	8.34	38		
58	0.013	-1.2	Clusterin	3	P10909	17	5.88	52	5.1	34
			Inter-alpha-trypsin inhibitor heavy chain H4	6	Q14624	12	6.51	103		
59	0.008	1.2	Complement factor B	16	P00751	46	6.67	86	6.3	85
			Plasma protease C1 inhibitor	6	P05155	24	6.09	55		
			Complement component C7	8	P10643	26	6.09	94		
			Complement C2	6	P06681	15	7.23	83		
			Complement C4-A; (B)	4	P0C0L4;P0C0L5	6	6.66;6.73	193;193		
			Gelsolin	2	P06396	5	5.90	86		
60	0.01	1.2	Inter-alpha-trypsin inhibitor heavy chain H4	24	Q14624	47	6.51	103	5.1	110
			Inter-alpha-trypsin inhibitor heavy chain H1	6	P19827	17	6.31	101		
			Complement C5	2	P01031	3	6.11	188		
			Phosphatidylinositol-glycan-specific phospholipase D	4	P80108	12	5.91	92		
			Complement C4-A; (B)	2	P0C0L4;P0C0L5	5	6.66;6.73	193;193		
			Angiotensinogen	3	P01019	18	5.87	53		
			Prothrombin	2	P00734	15	5.63	70		
			Afamin	2	P43652	11	5.64	69		
61	0.013	1.2	Inter-alpha-trypsin inhibitor heavy chain H4	19	Q14624	38	6.51	103	5.4	110
			Ceruloplasmin	10	P00450	25	5.44	122		
			Antithrombin-III	5	P01008	20	6.32	53		
			Insulin-like growth factor-binding protein complex acid labile subunit	2	P35858	11	6.33	66		
			Serum albumin	2	P02768	7	5.92	69		
			Inter-alpha-trypsin inhibitor heavy chain H3	2	Q06033	5	5.49	100		

p - ANOVA p-value; **fold** - fold difference (multiplication) when RCMD patients and healthy controls compared (+fold when the normalized volumes increased in RCMD); **protein** - protein identification; **peptides** - number of unique peptides fulfilling a minimal Mascot score; **AC** - accession number (SWISS-PROT); **SC** - protein sequence coverage; **pI** and **Mw** - calculated and experimental pI and molecular weight values
