# Supplementary material for: Plasma proteome changes associated with refractory cytopenia with multilineage dysplasia
Source: Proteome Sci. 2011 Oct 5;9:64. doi: 10.1186/1477-5956-9-64 (PMC3192726; doi:10.1186/1477-5956-9-64)
Supplement: Additional file 2 — Figure S1 - The peptide sequences corresponding to the perlecan fragment endorepellin. An illustration of the coverage of perlecan sequence (shown in bold red) that corresponds to the perlecan fragment endorepellin (3687-4391 amino acid sequence of perlecan) or more precisely to the perlecan/endorepellin fragment LG3 (4197-4391 amino acid sequence of perlecan). The MS/MS spectra of three peptides fulfilling a minimal Mascot score for identity 4236TSTASGLLLWQGVEVGEAGQGK4257, 4258DFISLGLQDGHLVFR4272, and 4330GSVYIGGAPDVATLTGGR4347 are shown. [file 1477-5956-9-64-S2.PDF]

Match to: **PGBM_HUMAN** Score: **139**
Basement membrane-specific heparan sulfate proteoglycan core protein OS=Homo sapiens GN=HSPG2 PE=1 SV=3
Found in search of DATA.TXT

Nominal mass (M_r): **479221**; Calculated pI value: **6.06**
NCBI BLAST search of PGBM_HUMAN against nr
Unformatted sequence string for pasting into other applications

Taxonomy: Homo sapiens

Fixed modifications: Carbamidomethyl (C)
Variable modifications: Oxidation (M)
Cleavage by Trypsin: cuts C-term side of KR unless next residue is P
Sequence Coverage: **1%**

Matched peptides shown in **Bold Red**

1 MGWRAAGALL LALLLHGRLL AVTHGLRAYD GLSLPEDIET VTASQMRWTH
51 SYLSDDEDML ADSISGDDLG SGDLGSGDFQ MVYFRALVNF TRSIEYSPQL
101 EDAGSREFRE VSEAVVDTLE SEYLKIPGDQ VVSVVFIKEL DGWVFVELDV
151 GSEGNADGAQ IQEMLLRVIS SGSVASYVTS PQGFQFRRLG TVPQFPRACT
201 EAEFACHSYN ECVALEYRCD RRPDCRDMSD ELNCEEPVLG ISPTFSLLVE
251 TTSLPPRPET TIMRQPPVTH APQPLLPGSV RPLPCGPQEA ACRNGHCIPR
301 DYLCDGQEDC EDGSDELDCG PPPPCEPNEF PCGNGHCALK LWRCDGDFDC
351 EDRTDEANCP TKRPEEVCGP TQFRCVSTNM CIPASFHCDE ESDCPDRSDE
401 FGCMPPQVVT PPRESIQASR GQTVTFTCVA IGVPTPIINW RLNWGHIPSH
451 PRVTVTSEGG RGTLIIRDVK ESDQGAYTCE AMNARGMVFG IPDGVLELVP
501 QRGPCPDGHF YLEHSAACLP CFCFGITSVC QSTRRFRDQI RLRFDQPDDF
551 KGVNVTMPAQ PGTPPLSSTQ LQIDPSLHEF QLVDLSRRFL VHDSFWALPE
601 QFLGNKVDSY GGSLRYNVRY ELARGMLEPV QRPDVVLVGA GYRLLSRGHT
651 PTQPGALNQR QVQFSEEHWV HESGRPVQRA ELLQVLQSLE AVLIQTVYNT
701 KMASVGLSDI AMDTTVTHAT SHGRAHSVEE CRCPIGYSGL SCESCDAHFT
751 RVPGGPYLGT CSGCNCNGHA SSCDPVYGHC LNCQHNTEGP QCNKCKAGFF
801 GDAMKATATS CRPCPCPYID ASRRFSDTCF LDTDGQATCD ACAPGYTGRR
851 CESCAPGYEG NPIQPGGKCR PVNQEIVRCD ERGSMGTSGE ACRCKNNVVG
901 RLCNECADGS FHLSTRNPDG CLKCFCMGVS RHCTSSSWSR AQLHGASEEP
951 GHFSLTNAAS THTTNEGIFS PTPGELGFSS FHRLLSGPYF WSLPSRFLGD
1001 KVTSYGGELR FTVTQRSQPG STPLHGQPLV VLQGNNIILE HHVAQEPSPG
1051 QPSTFIVPFR EQAWQRPDGQ PATREHLLMA LAGIDTLLIR ASYAQQPAES
1101 RVSGISMDVA VPEETGQDPA LEVEQCSCPP GYRGPSCQDC DTGYTRTPSG
1151 LYLGTCERCS CHGHSEACEP ETGACQGCQH HTEGPRCEQC QPGYYGDAQR
1201 GTPQDCQLCP CYGDPAAGQA AHTCFLDTDG HPTCDACSPG HSGRHCERCA
1251 PGYYGNPSQG QPCQRDSQVP GPIGCNCDPQ GSVSSQCDAA GQCQCKAQVE
1301 GLTCSHCRPH HFHLSASNPD GCLPCFCMGI TQQCASSAYT RHLISTHFAP
1351 GDFQGFALVN PQRNSRLTGE FTVEPVPEGA QLSFGNFAQL GHESFYWQLP
1401 ETYQGDKVAA YGGKLRYTLS YTAGPQGSPL SDPDVQITGN NIMLVASQPA
1451 LQGPERRSYE IMFREEFWRR PDGQPATREH LLMALADLDE LLIRATFSSV
1501 PLAASISAVS LEVAQPGPSN RPRALEVEEC RCPPGYIGLS CQDCAPGYTR
1551 TGSGLYLGHC ELCECNGHSD LCHPETGACS QCQHNAAGEF CELCAPGYYG
1601 DATAGTPEDC QPCACPLTNP ENMFSRTCES LGAGGYRCTA CEPGYTGQYC
1651 EQCGPGYVGN PSVQGGQCLP ETNQAPLVVE VHPARSIVPQ GGSHSLRCQV
1701 SGSPPHYFYW SREDGRPVPS GTQQRHQGSE LHFPSVQPSD AGVYICTCRN
1751 LHQSNTSRAE LLVTEAPSKP ITVTVEEQRS QSVRPGADVT FICTAKSKSP
1801 AYTLVWTRLH NGKLPTRAMD FNGILTIRNV QLSDAGTYVC TGSNMFAMDQ
1851 GTATLHVQAS GTLSAPVVSI HPPQLTVQPG QLAEFRCSAT GSPTPTLEWT
1901 GGPGGQLPAK AQIHGGILRL PAVEPTDQAQ YLCRAHSSAG QQVARAVLHV
1951 HGGGGPRVQV SPERTQVHAG RTVRLYCRAA GVPSATITWR KEGGSLPPQA
2001 RSERTDIATL LIPAITTADA GFYLCVATSP AGTAQARIQV VVLSASDASP
2051 PPVKIESSSP SVTEGQTLDL NCVVAGSAHA QVTWYRRGGS LPPHTQVHGS
2101 RLRLPQVSPA DSGEYVCRVE NGSGPKEASI TVSVLHGTHS GPSYTPVPGS
2151 TRPIRIEPSS SHVAEGQTLD LNCVVPGQAH AQVTWHKRGG SLPARHQTHG
2201 SLLRLHQVTP ADSGEYVCHV VGTSGPLEAS VLVTIEASVI PGPIPPVRIE
2251 SSSSTVAEGQ TLDLSCVVAG QAHAQVTWYK RGGSLPARHQ VRGSRLYIFQ
2301 ASPADAGQYV CRASNGMEAS ITVTVTGTQG ANLAYPAGST QPIRIEPSSS
2351 QVAEGQTLDL NCVVPGQSHA QVTWHKRGGS LPVRHQTHGS LLRLYQASPA
2401 DSGEYVCRVL GSSVPLEASV LVTIEPAGSV PALGVTPTVR IESSSSQVAE
2451 GQTLDLNCLV AGQAHAQVTW HKRGGSLPAR HQVHGSRLRL LQVTPADSGE
2501 YVCRVVGSSG TQEASVLVTI QQRLSGSHSQ GVAYPVRIES SSASLANGHT
2551 LDLNCLVASQ APHTITWYKR GGSLPSRHQI VGSRLRIPQV TPADSGEYVC
2601 HVSNGAGSRE TSLIVTIQGS GSSHVPSVSP PIRIESSSPT VVEGQTLDLN
2651 CVVARQPQAI ITWYKRGGSL PSRHQTHGSH LRLHQMSVAD SGEYVCRANN
2701 NIDALEASIV ISVSPSAGSP SAPGSSMPIR IESSSSHVAE GETLDLNCVV
2751 PGQAHAQVTW HKRGGSLPSH HQTRGSRLRL HHVSPADSGE YVCRVMGSSG
2801 PLEASVLVTI EASGSSAVHV PAPGGAPPIR IEPSSSRVAE GQTLDLKCVV
2851 PGQAHAQVTW HKRGGNLPAR HQVHGPLLRL NQVSPADSGE YSCQVTGSSG
2901 TLEASVLVTI EPSSPGPIPA PGLAQPIYIE ASSSHVTEGQ TLDLNCVVPG
2951 QAHAQVTWYK RGGSLPARHQ THGSQLRLHL VSPADSGEYV CRAASGPGPE
3001 QEASFTVTVP PSEGSSYRLR SPVISIDPPS STVQQGQDAS FKCLIHDGAA
3051 PISLEWKTRN QELEDNVHIS PNGSIITIVG TRPSNHGTYR CVASNAYGVA
3101 QSVVNLSVHG PPTVSVLPEG PVWVKVGKAV TLECVSAGEP RSSARWTRIS
3151 STPAKLEQRT YGLMDSHAVL QISSAKPSDA GTYVCLAQNA LGTAQKQVEV
3201 IVDTGAMAPG APQVQAEEAE LTVEAGHTAT LRCSATGSPA PTIHWSKLRS
3251 PLPWQHRLEG DTLIIPRVAQ QDSGQYICNA TSPAGHAEAT IILHVESPPY
3301 ATTVPEHASV QAGETVQLQC LAHGTPPLTF QWSRVGSSLP GRATARNELL
3351 HFERAAPEDS GRYRCRVTNK VGSAEAFAQL LVQGPPGSLP ATSIPAGSTP
3401 TVQVTPQLET KSIGASVEFH CAVPSDRGTQ LRWFKEGGQL PPGHSVQDGV
3451 LRIQNLDQSC QGTYICQAHG PWGKAQASAQ LVIQALPSVL INIRTSVQTV
3501 VVGHAVEFEC LALGDPKPQV TWSKVGGHLR PGIVQSGGVV RIAHVELADA
3551 GQYRCTATNA AGTTQSHVLL LVQALPQISM PQEVRVPAGS AAVFPCIASG
3601 YPTPDISWSK LDGSLPPDSR LENNMLMLPS VRPQDAGTYV CTATNRQGKV
3651 KAFAHLQVPE RVVPYFTQTP YSFLPLPTIK DAYRKFEIKI TFRPDSADGM
3701 LLYNGQKRVP GSPTNLANRQ PDFISFGLVG GRPEFRFDAG SGMATIRHPT
3751 PLALGHFHTV TLLRSLTQGS LIVGDLAPVN GTSQGKFQGL DLNEELYLGG
3801 YPDYGAIPKA GLSSGFIGCV RELRIQGEEI VFHDLNLTAH GISHCPTCRD
3851 RPCQNGGQCH DSESSSYVCV CPAGFTGSRC EHSQALHCHP EACGPDATCV
3901 NRPDGRGYTC RCHLGRSGLR CEEGVTVTTP SLSGAGSYLA LPALTNTHHE
3951 LRLDVEFKPL APDGVLLFSG GKSGPVEDFV SLAMVGGHLE FRYELGSGLA
4001 VLRSAEPLAL GRWHRVSAER LNKDGSLRVN GGRPVLRSSP GKSQGLNLHT
4051 LLYLGGVEPS VPLSPATNMS AHFRGCVGEV SVNGKRLDLT YSFLGSQGIG
4101 QCYDSSPCER QPCQHGATCM PAGEYEFQCL CRDGFKGDLC EHEENPCQLR
4151 EPCLHGGTCQ GTRCLCLPGF SGPRCQQGSG HGIAESDWHL EGSGGNDAPG
4201 QYGAYFHDDG FLAFPGHVFS R**SLPEVPETI ELEVRTSTAS GLLLWQGVEV**
4251 **GEAGQGKDFI SLGLQDGHLV FR**YQLGSGEA RLVSEDPIND GEWHRVTALR
4301 EGRRGSIQVD GEELVSGRSP GPNVAVNAK**G SVYIGGAPDV ATLTGGR**FSS
4351 GITGCVKNLV LHSARPGAPP PQPLDLQHRA QAGANTRPCP S

4236 - 4257 TSTASGLLLWQGVEVGEAGQGK

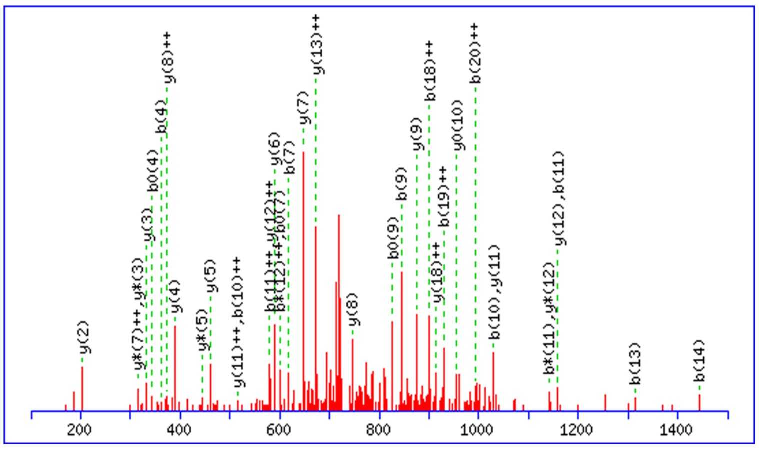

4258 - 4272 DFISLGLQDGHLVFR

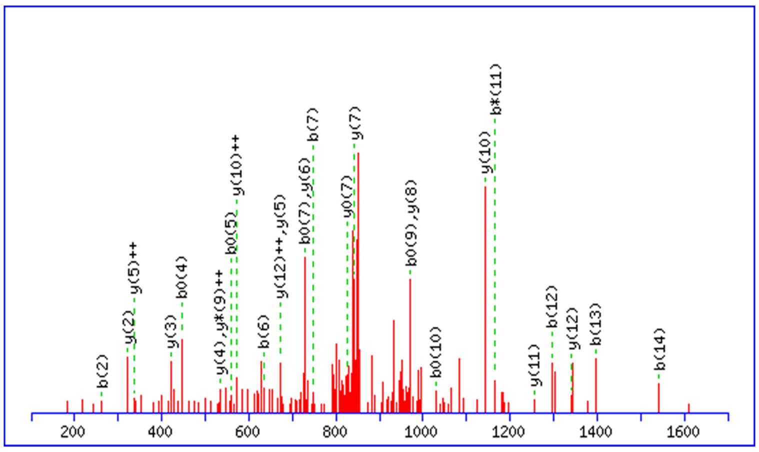

4330 - 4347 GSVYIGGAPDVATLTGGR

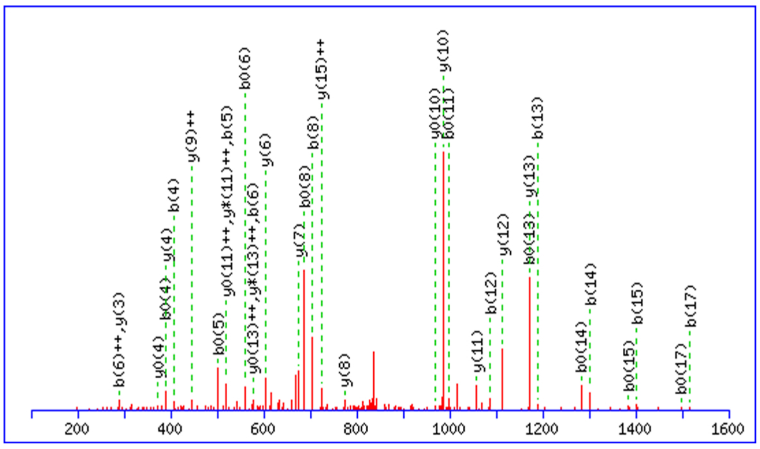

Show predicted peptides also

Sort Peptides By ◉ Residue Number ○ Increasing Mass ○ Decreasing Mass

Start - End	Observed	Mr(expt)	Mr(calc)	%	Miss	Sequence
4222 - 4235	537.7000	1610.0782	1609.8563	0.01378	0	R.SLPEVPETIELEVR.T (Ions score 19)
4236 - 4257	730.5000	2188.4782	2187.1172	0.06223	0	R.TSTASGLLLWQGVEVGEAGQGK.D (Ions score 52)
4258 - 4272	858.1000	1714.1854	1715.8995	-0.09989	0	K.DFISLGLQDGHLVFR.Y (Ions score 56)
4258 - 4272	572.7000	1715.0782	1715.8995	-0.04787	0	K.DFISLGLQDGHLVFR.Y (Ions score 48)
4258 - 4272	572.9000	1715.6782	1715.8995	-0.01290	0	K.DFISLGLQDGHLVFR.Y (Ions score 29)
4330 - 4347	846.0000	1689.9854	1689.8686	0.00691	0	K.GSVYIGGAPDVATLTGGR.F (Ions score 59)
